# Aligned and discordant profiles of nursing informatics competency and information-security attitudes among clinical nurses: a single-centre study in China

**DOI:** 10.3389/fmed.2026.1987978

**Published:** 2026-10-14

**Authors:** Xiwei Wang, Qiongfang Wang, Dongxia Cao

**Affiliations:** 1 Department of Gastroenterology, Sichuan Provincial People’s Hospital, University of Electronic Science and Technology of China, Chengdu, Sichuan, China; 2 School of Medicine, University of Electronic Science and Technology of China, Chengdu, Sichuan, China

**Keywords:** clinical nurses, hospital ethical climate, information-security attitudes, latent profile analysis, nurse–patient relationship, nursing informatics competency, patient information security

## Abstract

**Background:**

Secure digital nursing practice requires both nursing informatics competency and favourable information-security attitudes, yet these domains may not develop in parallel within individual nurses. This study aimed to identify joint profiles of nursing informatics competency and information-security attitudes among clinical nurses, examine characteristics associated with profile membership, and compare nurse-perceived nurse–patient relationship quality across profiles.

**Methods:**

A single-centre cross-sectional survey was conducted among a convenience sample of 1,149 clinical nurses at a tertiary hospital in China between February and June 2026. Latent profile analysis used nine standardised competency and attitude dimensions. Three-step analyses examined profile correlates and differences in nurse-perceived nurse–patient relationship quality. A complementary configurational analysis examined combinations associated with high and non-high relationship quality.

**Results:**

Six competency–attitude profiles were identified, comprising aligned low and high patterns, two contrasting discordant patterns and two mixed patterns. A more favourable perceived hospital ethical climate was consistently associated with the high competency–high attitude profile, which retained the highest adjusted nurse-perceived nurse–patient relationship quality, although between-profile differences were attenuated after adjustment. High competency–high attitude profile membership combined with a favourable perceived hospital ethical climate formed the core configuration associated with high relationship quality.

**Conclusion:**

Nursing informatics competency and information-security attitudes do not necessarily develop in parallel. Joint assessment may reveal combinations of strengths and support needs obscured when the domains are considered separately, and these findings may inform more differentiated professional development and workplace support for secure digital nursing practice.

## 1 Introduction

Digital technologies, including electronic health records, hospital information systems and mobile clinical applications, are now embedded in routine nursing practice, reshaping how nurses access, document, communicate and manage patient information. Recent large-scale breaches of protected health information have underscored the continuing vulnerability of digitally stored and exchanged patient data (1). As nurses increasingly work through digital systems, protecting patient information has become not only a technical and regulatory requirement but also an integral component of professional nursing practice. Regulatory and policy developments across jurisdictions increasingly emphasize the protection of sensitive health information and organisational responsibilities for secure information handling, monitoring and incident response (2–4). Failures in secure information practice can affect not only confidentiality and regulatory compliance but also professional accountability and patient trust. Accordingly, readiness for secure digital practice is increasingly part of the professional capabilities required of nurses in contemporary healthcare.

Effective digital nursing therefore requires both the capability to work with clinical information technologies and an appropriate orientation towards confidentiality, access control and secure information handling. Nursing informatics competency (NIC) and information-security attitudes (ISA) represent complementary but distinct domains relevant to nurses’ readiness for secure patient-information handling (5, 6). However, sample-level associations between NIC and ISA may not capture how these domains coexist within individual nurses. Nurses with similar overall levels in one domain may differ substantially in the other, giving rise to distinct within-person competency–attitude configurations.

Previous research has largely examined NIC and ISA separately or as individual factors associated with information-security behaviour (7). Person-centred studies have identified distinct NIC profiles among registered and clinical nurses (8, 9), while latent-class research has likewise demonstrated heterogeneity in ISA among clinical nurses (10). Variable-centred evidence further indicates that higher NIC is associated with more favourable ISA (11). However, it remains unclear how NIC and ISA are configured jointly within the same nurse. Existing studies therefore provide limited insight into whether distinct subgroups are characterised by aligned or discordant cross-domain patterns. A joint person-centred analysis extends this literature by modelling the two domains simultaneously and identifying subgroups defined by their combined competency–attitude configurations.

Such competency–attitude patterns also need to be considered within the organisational and workplace context in which nurses develop and enact digital practice. Social Cognitive Theory provides an organising lens for considering the interplay of personal and environmental factors (12). Within this framework, NIC was conceptualised as a capability-related personal factor, ISA as a cognitive and attitudinal factor, and perceived hospital ethical climate as an environmental factor reflecting the organisational context in which secure information handling occurs. Ethical climate reflects employees’ perceptions of organisational norms and relationships relevant to ethical practice and has been associated with nursing and work-related outcomes (13, 14). More proximal and potentially modifiable workplace resources—including information-security or cybersecurity training, familiarity with patient-information protection policies and knowledge of incident-reporting procedures—may also be relevant to how nurses understand and enact information-security responsibilities (7). The reported frequency of information-security risk events may provide an additional indication of the security challenges nurses encounter in everyday digital practice. Examining these factors in relation to profile membership may help distinguish the broader perceived ethical climate from more proximal workplace resources associated with different competency–attitude patterns and identify contextual factors that may be relevant to differentiated professional development and support.

Nurse-perceived nurse–patient relationship quality was included because confidentiality, professional accountability and trust are integral to professional nurse–patient interactions (15). Electronic health records and other digital systems can influence nurses’ attention, communication and interaction with patients (16), suggesting that relational quality may differ across competency–attitude configurations. Comparing nurse–patient relationship quality across profiles may therefore help assess whether the identified configurations have relevance beyond statistical classification. However, relational quality may also reflect the combined influence of individual and contextual conditions. A complementary configurational analysis was therefore used to examine whether different combinations of profile membership, perceived hospital ethical climate, workplace-related factors and information-security risk-event frequency were associated with high or non-high nurse-perceived nurse–patient relationship quality. Given its multifactorial nature, nurse–patient relationship quality was treated as a distal relational outcome rather than a direct consequence of profile membership.

Accordingly, this study aimed to identify joint profiles of nursing informatics competency and information-security attitudes among clinical nurses, examine factors associated with profile membership, and compare nurse-perceived nurse–patient relationship quality across the identified profiles. Individual, workplace-related and perceived organisational characteristics were considered as potential profile correlates. In addition, a complementary exploratory configurational analysis examined combinations of profile membership, perceived hospital ethical climate, workplace-related factors and information-security risk-event frequency associated with high and non-high nurse-perceived nurse–patient relationship quality.

## 2 Materials and methods

### 2.1 Study design and participants

This single-centre cross-sectional survey was conducted among clinical nurses from February to June 2026 at a tertiary Grade A hospital in Sichuan Province, China. Participants were recruited using convenience sampling. Eligible participants were registered nurses with at least 6 months of frontline clinical experience who routinely used hospital information systems or handled patient information, provided informed consent, and completed the questionnaire independently. Interns, visiting nurses, and nurses not engaged in frontline clinical practice during the survey period were excluded. Sample-size adequacy was considered with reference to methodological evidence specific to latent profile analysis rather than an item-to-participant ratio. Simulation studies indicate that correct profile enumeration depends jointly on sample size, the number of indicators and profiles, and the degree of between-profile separation (17). The study was reported in accordance with the Strengthening the Reporting of Observational Studies in Epidemiology (STROBE) statement (18).

### 2.2 Data collection

The electronic questionnaire was administered through Wenjuanxing (https://www.wjx.cn/) and distributed via nursing WeChat groups. Under this recruitment approach, the total number of nurses who received the survey invitation or met the eligibility criteria could not be reliably ascertained, precluding calculation of a conventional response rate. All items were mandatory, resulting in no item-level missing data. For data-quality control, completion time was used as a data-quality indicator (19, 20), and the 240-s cutoff was defined based on questionnaire length and the empirical distribution of completion times. Submissions completed in <240 s or same-IP duplicates with identical responses across all 90 scale items were excluded, with only the first eligible response retained. Of the 191 excluded questionnaires, 22 were from respondents who did not agree to participate, 117 were completed in <240 s, and 52 were duplicate submissions.

### 2.3 Measures

A researcher-developed questionnaire collected demographic and professional characteristics together with information-security-related variables, including dedicated information-security/cybersecurity training, familiarity with institutional patient-information protection policies, knowledge of information-security incident-reporting procedures, and self-reported information-security risk-event experience. The exact response categories are presented in Table 1. Training was coded as 0 = no and 1 = yes; policy familiarity and reporting-procedure knowledge were coded from 1 to 4 in ascending order; and risk-event experience was treated as a five-category variable, with “never” as the reference category in regression analyses. In the questionnaire, information-security risk events were described to participants as patient-information transmission risks or near-miss events, with examples including sending information to the wrong recipient, incorrect transmission, unauthorized viewing, and external sharing of screenshots.

**Table 1:** Participant characteristics (*n* = 1,149).

Characteristic	Category	*n*	%
Sex	Female	1,082	94.2
	Male	67	5.8
Age (years)	≤25	86	7.5
	26–30	242	21.1
	31–35	291	25.3
	36–40	250	21.8
	>40	280	24.4
Education	Junior college or below	211	18.4
	Bachelor's degree	896	78.0
	Master's degree or above	42	3.7
Years of clinical experience	<1 year	38	3.3
	1–5 years	197	17.1
	6–10 years	237	20.6
	11–15 years	291	25.3
	16–20 years	158	13.8
	>20 years	228	19.8
Professional title	Nurse	151	13.1
	Senior nurse	376	32.7
	Supervisor nurse	500	43.5
	Associate chief nurse or above	122	10.6
Position	Staff nurse	753	65.5
	Nursing team leader/clinical instructor	204	17.8
	Head nurse	157	13.7
	Other	35	3.0
Department	Internal medicine	383	33.3
	Surgery (including orthopedics)	245	21.3
	Obstetrics and gynecology	67	5.8
	Pediatrics (including neonatal units)	72	6.3
	Emergency department	27	2.3
	Intensive care unit	16	1.4
	Operating room	51	4.4
	Outpatient/day ward	78	6.8
	Other	210	18.3
Information-security/cybersecurity training	Yes	988	86.0
	No	161	14.0
Familiarity with patient-information protection policies	Not familiar	10	0.9
	Slightly familiar	199	17.3
	Fairly familiar	489	42.6
	Very familiar	451	39.3
Knowledge of the information-security incident-reporting procedure	Unclear	82	7.1
	Somewhat clear	233	20.3
	Clear	418	36.4
	Very clear	416	36.2
Self-reported information-security risk-event frequency	Never	712	62.0
	Occasionally	268	23.3
	Sometimes	105	9.1
	Frequently	20	1.7
	Unsure/prefer not to answer	44	3.8

Percentages may not sum to 100.0% because of rounding. Open-text responses for position and department were consolidated into the “Other” category; orthopedics was classified under surgery, and neonatal units under pediatrics.

#### 2.3.1 Nursing informatics competency

Nursing informatics competency (NIC) was assessed using the 26-item Nursing Informatics Competency Assessment Scale for Clinical Nurses (21), comprising computer competence, information competence and comprehensive application competence. Items are rated on a 5-point scale (total score 26–130), with higher scores indicating greater NIC. Cronbach's *α* was 0.977 in the present study.

#### 2.3.2 Information-security attitudes

Information-security attitudes (ISA) were assessed using the 30-item Chinese version of the Information Security Attitude Questionnaire for Nurses, originally developed by Kang and Seomun (5) and psychometrically evaluated in Chinese by Liu et al. (22). The scale comprises environmental control, facility stability, access restriction, work systematization, professional responsibility and continuing education. Items are rated on a 4-point scale (total score 30–120), with higher scores indicating more favourable ISA. Cronbach's *α* was 0.968. Dimension-specific Cronbach's *α* coefficients for the three NIC and six ISA dimensions used as LPA indicators, together with their Pearson intercorrelations, are reported in Supplementary Table S7.

#### 2.3.3 Perceived hospital ethical climate

Perceived hospital ethical climate (HEC) was assessed using the 25-item Chinese Hospital Ethical Climate Survey, originally developed by Olson (23) and adapted by Wang (24). Items are rated on a 5-point scale (total score 25–125), with higher scores indicating a more favourable perceived ethical climate. Cronbach's *α* was 0.968. HEC was analysed as an individual-level perception rather than an aggregated organisational measure.

#### 2.3.4 Nurse-perceived nurse–patient relationship quality

Nurse-perceived nurse–patient relationship quality (NPR) was assessed using the 9-item Nurse–Patient Relationship Scale in China (25). Items are rated on a 6-point scale (total score 9–54), with higher scores indicating better relationship quality. The measure reflects nurses’ rather than patients’ perceptions. Cronbach's *α* was 0.951.

### 2.4 Statistical analysis

Data preparation. Descriptive analyses were conducted using SPSS 27.0. The three NIC and six ISA dimensions used as profile indicators were standardised as z scores to place indicators measured on different response scales on a comparable metric.

#### 2.4.1 Joint latent profile analysis

LPA was conducted in Mplus 8.3 using robust maximum likelihood estimation. Profile means were freely estimated. Indicator-specific variances were estimated separately but constrained to equality across profiles to limit class-specific parameters and maintain a parsimonious model specification, whereas within-profile covariances were fixed at zero under the local-independence assumption; the restrictiveness of this assumption was examined in a sensitivity model (17, 26, 27). One- to eight-profile solutions were evaluated using AIC, BIC, aBIC, entropy, LMR-LRT, BLRT, profile size, stability, interpretability and parsimony. Model selection considered these criteria collectively rather than relying on any single fit index. Enumeration models used 1,000 initial-stage random starts and 250 final-stage optimisations, and the retained six-profile solution was re-estimated using 5,000 and 1,000 starts, respectively. Pearson correlations among the nine profile indicators were examined, and a fixed six-profile sensitivity model was estimated with four class-invariant within-profile residual covariances freely estimated to relax the local-independence assumption. Profile labels were descriptive and sample-relative, reflecting patterns in the z-standardised indicators rather than externally validated competency levels; they should not be interpreted as diagnostic categories or validated thresholds for workforce assessment. Full details and Mplus syntax are provided in the Supporting Information.

#### 2.4.2 Profile correlates and distal outcomes

R3STEP multinomial logistic regression examined individual, governance-related and perceived organisational characteristics associated with profile membership while accounting for classification uncertainty (28, 29). Covariates entered simultaneously were information-security/cybersecurity training, policy familiarity, reporting-procedure knowledge, information-security risk-event experience, perceived hospital ethical climate, professional title, years of clinical experience, age group, and educational level. Sex, position, and department were not included because of marked sex imbalance and sparse categories for position and department, which would have increased model complexity. Ordinal predictors were entered as ascending category scores to estimate a single linear trend across categories, assuming an approximately constant change in log odds between adjacent categories. Information-security risk-event frequency was treated as a five-category variable with “never” as the reference, and HEC was entered as the final 25-item HECS mean score, and its effects were expressed per 1-SD increase. BCH was used to compare NPR across profiles while accounting for classification uncertainty (30, 31), with pairwise *P* values adjusted using the Holm procedure (32). An additional covariate-adjusted BCH distal-outcome analysis examined whether profile differences in NPR persisted after accounting for perceived HEC, age group, educational level, years of clinical experience, and professional title, while retaining correction for classification uncertainty through BCH weights. Holm adjustment was applied to pairwise comparisons. To assess sensitivity to the NPR ceiling effect, DCAT was used to compare the probability of attaining a near-ceiling NPR score (≥53) across profiles. In the absence of a validated near-ceiling cutoff, ≥53 was defined operationally as the two highest attainable total scores (53–54) on the 9–54 scale, to capture observations concentrated at the upper boundary of the scale. Analyses excluding maximum-score observations provided an additional sensitivity check. Further details are provided in the Supporting Information.

#### 2.4.3 Complementary configurational analysis

An exploratory fsQCA was conducted in R 4.6.1 using the QCA package (version 3.25) to examine configurations associated with high and non-high NPR (33, 34). To complement the preceding profile-based analyses, fsQCA examined whether combinations of profile membership and selected contextual, governance-related, and experience-related conditions were sufficient in set-theoretic terms for high or non-high NPR, and whether the configurational patterns differed between the two outcomes. This approach addressed conjunctural sufficiency and outcome asymmetry rather than estimating additional net or interaction effects. Conditions included membership in the high competency–high attitude profile, perceived HEC, training, policy familiarity, reporting-procedure knowledge and low information-security risk-event frequency. Conditions and outcomes were calibrated into sets, followed by necessity and truth-table sufficiency analyses. For the continuous HECH and HNPR sets, primary direct-calibration anchors were based on the 90th percentile, median, and 10th percentile of the observed distributions for full membership, crossover, and full non-membership, respectively; for HNPR, the 90th percentile corresponded to the scale maximum of 54, and alternative P75/P50/P25 anchors were examined as part of the sensitivity analyses. Both parsimonious and intermediate solutions were examined, and solution consistency, PRI and coverage were evaluated. Detailed calibration procedures, directional expectations used for the intermediate solutions, truth-table thresholds and robustness analyses are reported in the Supporting Information.

### 2.5 Ethical considerations

Ethical approval was obtained from the Basic and Clinical Research Ethics Committee of Sichuan Academy of Medical Sciences & Sichuan Provincial People's Hospital (Approval No. 2026-527). The study was conducted in accordance with the Declaration of Helsinki. Participation was voluntary, and electronic informed consent was obtained from all participants before questionnaire completion. No names or other direct personal identifiers were collected. Data were stored securely, with access restricted to the research team.

## 3 Results

### 3.1 Participant characteristics

Of the 1,340 submitted questionnaires, 1,149 (85.75%) met the criteria for inclusion in the analysis. Most participants were women (94.2%), held a bachelor's degree (78.0%), and had received information-security or cybersecurity training (86.0%) (Table 1).

### 3.2 Joint profile identification

One- to eight-profile solutions were compared (Table 2). The six-profile solution showed high classification quality (entropy = 0.939), with a smallest profile of 61 nurses (5.31%). Although AIC, BIC, and aBIC continued to decrease and BLRT remained significant in the seven- and eight-profile solutions, the adjusted LMR-LRT was no longer significant for either the seven-profile (*P* = 0.204) or eight-profile solution (*P* = 0.510). The eight-profile solution also produced a very small profile (*n* = 7, 0.61%) and an estimation warning. The six-profile solution was stable under increased random starts. Although the seven-profile solution yielded lower information criteria, the adjusted LMR-LRT was not significant, entropy decreased slightly, and the smallest profile accounted for 4.79% of the sample. Moreover, the additional profile did not represent a clearly distinct substantive pattern. Considering these findings together with interpretability and parsimony, the six-profile solution was retained. With 5,000 initial-stage random starts and 1,000 final-stage optimisations, the best log-likelihood of the retained six-profile model (−9744.622) was replicated 215 times. Pearson correlations among the nine LPA indicators ranged from 0.267 to 0.842 (all *P* < .001; Supplementary Table S7). In a sensitivity analysis allowing four class-invariant within-profile residual covariances, the fixed six-profile model remained numerically stable (entropy = 0.916; smallest profile = 4.96%). Several major profile patterns remained recognisable, although the distinction between C1 and C3 was attenuated and C6 showed some redistribution. Thus, the profile structure showed partial rather than full robustness to relaxation of the local-independence assumption (Supplementary Table S8).

**Table 2:** Fit statistics for latent profile solutions.

Profiles	Log-likelihood	AIC	BIC	aBIC	Entropy	LMR-LRT P	BLRT P	Min. profile *n* (%)	Decision
1	−14673.243	29382.487	29473.326	29416.153	—	—	—	1149 (100.00%)	
2	−11317.996	22691.993	22833.299	22744.362	0.984	<0.001	<0.001	440 (38.29%)	
3	−10727.214	21530.428	21722.200	21601.500	0.968	0.008	<0.001	156 (13.58%)	
4	−10291.742	20679.483	20921.722	20769.259	0.927	<0.001	<0.001	142 (12.36%)	
5	−9971.004	20058.008	20350.713	20166.487	0.941	0.016	<0.001	62 (5.40%)	
6	−9744.622	19625.244	19968.416	19752.427	0.939	0.006	<0.001	61 (5.31%)	Retained
7	−9577.546	19311.091	19704.730	19456.977	0.932	0.204	<0.001	55 (4.79%)	
8^a^	−9434.461	19044.922	19489.027	19209.511	0.937	0.510	<0.001	7 (0.61%)	

AIC, Akaike information criterion; aBIC, sample-size-adjusted Bayesian information criterion; BIC, Bayesian information criterion; BLRT, bootstrap likelihood ratio test; LMR-LRT, adjusted Lo–Mendell–Rubin likelihood ratio test. Model selection considered statistical fit, classification quality, profile size, stability, interpretability, and parsimony.

a The eight-profile solution produced a non-positive definite first-order derivative product matrix; some standard errors may therefore be unreliable.

### 3.3 Joint profile characteristics

The six-profile solution comprised two broadly aligned profiles, two opposing discordant profiles and two additional mixed profiles (Figure 1). C1 (low competency–low attitude; *n* = 183, 15.93%) and C4 (high competency–high attitude; *n* = 408, 35.51%) represented broadly aligned patterns, whereas C3 (attitude-lagged, competency-preserved; *n* = 139, 12.10%) and C6 (attitude-advantaged, competency-lagged; *n* = 262, 22.80%) represented opposing forms of discordance. C2 (competency-lagged, attitude-mixed; *n* = 61, 5.31%) and C5 (continuing-education-oriented lower profile; *n* = 96, 8.36%) showed additional mixed patterns. The average posterior classification probabilities were 0.957, 0.941, 0.950, 0.968, 0.964, and 0.933 for C1–C6, respectively. Exact standardized means for all nine indicators are provided in Supplementary Table S5.

**Figure 1:**
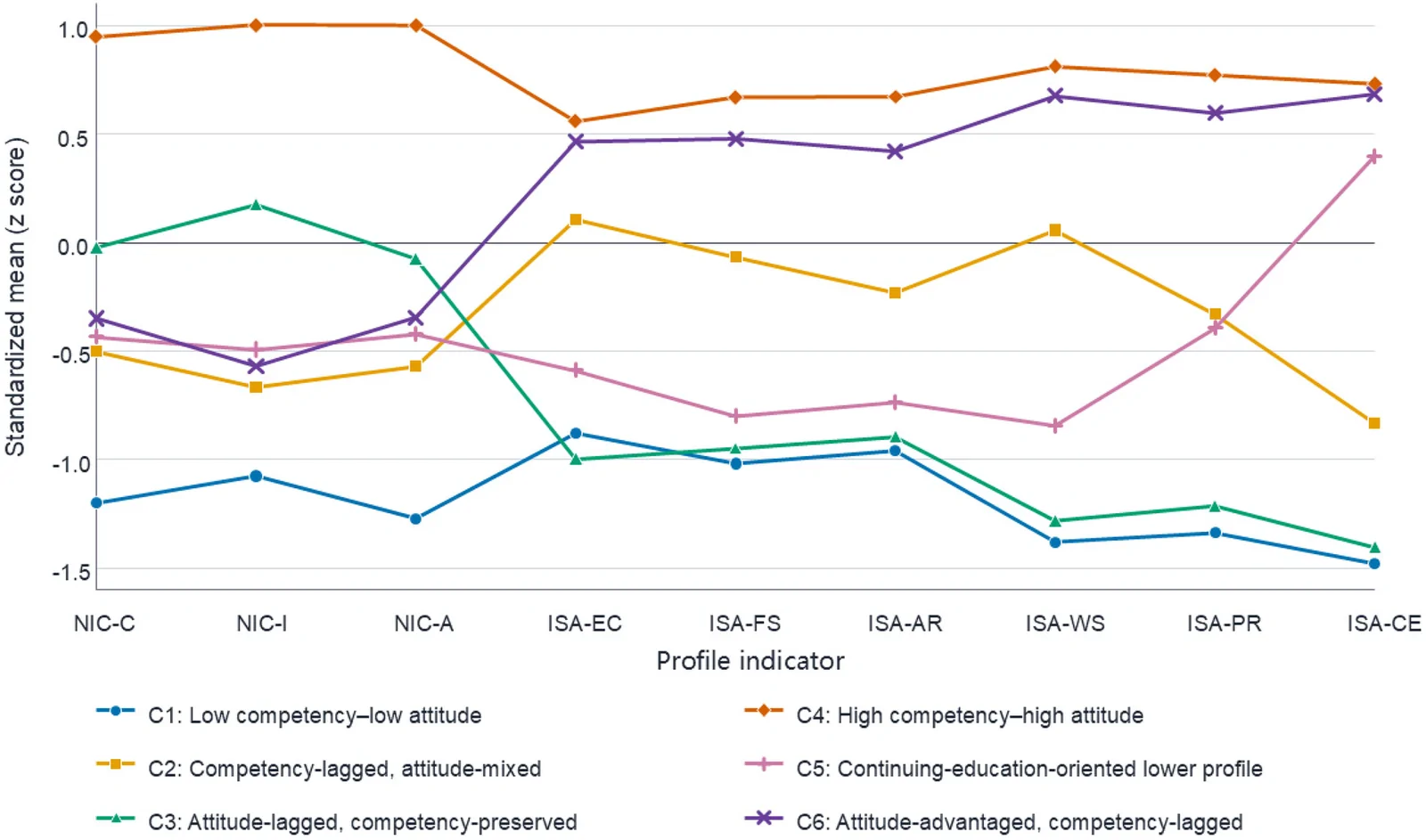
Standardised indicator patterns across six competency–attitude profiles. Values are profile-specific z-standardised means (0 = sample mean). NIC-C,  computer competence; NIC-I, information competence; NIC-A, comprehensive application competence; ISA-EC, environmental control; ISA-FS, facility stability; ISA-AR, access restriction; ISA-WS, work systematization; ISA-PR, professional responsibility; ISA-CE, continuing education. Profile labels reflect relative patterns within this sample.

### 3.4 Characteristics associated with profile membership

Spearman correlations among NIC, ISA, HEC, and NPR ranged from 0.549 to 0.746 (all *P* < .001). HEC was correlated with NIC (rₛ = 0.672), ISA (rₛ = 0.620), and NPR (rₛ = 0.746) (Supplementary Table S6). C4 had the highest perceived HEC scores, whereas the other profiles showed lower mean and median values, with overlapping score distributions across profiles (Table 3). Each 1-SD increase in perceived HEC was associated with lower odds of membership in C1, C2, C3, C5, and C6 relative to C4 (high competency–high attitude) (OR range, 0.035–0.153; all *P* < .001). Other covariates showed less consistent profile-specific associations; full R3STEP estimates are provided in the Supporting Information.

**Table 3:** Perceived hospital ethical climate across profiles.

Profile	HECS item mean ± SD	HECS item median (IQR)	OR (95% CI) vs. C4
C1	3.895 ± 0.438	3.96 (3.76–4.08)	0.035 (0.022–0.055)
C2	4.141 ± 0.365	4.04 (3.88–4.36)	0.081 (0.051–0.131)
C3	4.175 ± 0.371	4.04 (3.96–4.30)	0.091 (0.060–0.137)
C4	4.819 ± 0.301	4.96 (4.76–5.00)	Reference
C5	4.228 ± 0.349	4.16 (3.92–4.52)	0.116 (0.077–0.175)
C6	4.412 ± 0.444	4.44 (4.04–4.80)	0.153 (0.104–0.225)

C4 (high competency–high attitude) was the reference profile. HECS descriptive values are mean item scores on the 1–5 response scale. Odds ratios are reported per 1-SD increase in the final 25-item HECS mean score; all *P* < .001. Full R3STEP estimates for all covariates are provided in the Supporting Information.

### 3.5 Nurse–patient relationship quality across profiles

Overall, 490 participants (42.6%) had near-ceiling NPR scores (≥53), including 425 (37.0%) who attained the maximum score of 54. After adjustment for perceived HEC, age group, educational level, years of clinical experience, and professional title, NPR differed across profiles (Wald *χ*^2^(5) = 18.650, *P* = .002). C4 had the highest adjusted mean and, after Holm correction, remained higher than C2, C3, C5, and C6 but not C1. The unadjusted BCH comparison, reported for descriptive context, also differed across profiles (Wald *χ*^2^(5) = 601.220, *P* < .001), with C4 showing the highest mean. Ceiling-effect sensitivity analyses are reported in Table 4 and the Supporting Information.

**Table 4:** Nurse–patient relationship quality across profiles.

Profile	Adjusted BCH mean (95% CI)	Unadjusted BCH mean (95% CI)	DCAT probability of NPR ≥53 (95% CI)
C1	49.198 (48.186–50.211)	43.826 (42.842–44.810)	0.086 (0.043–0.129)
C2	47.811 (46.376–49.245)	46.618 (45.485–47.751)	0.116 (0.022–0.210)
C3	48.652 (47.941–49.362)	46.874 (46.053–47.695)	0.212 (0.139–0.285)
C4	50.814 (49.817–51.812)	52.767 (52.469–53.065)	0.802 (0.757–0.847)
C5	48.374 (47.470–49.278)	47.513 (46.596–48.430)	0.157 (0.079–0.235)
C6	49.110 (48.604–49.615)	48.918 (48.256–49.580)	0.367 (0.304–0.430)

BCH estimates account for classification uncertainty. Adjusted BCH estimates additionally account for perceived hospital ethical climate, age group, educational level, years of clinical experience, and professional title. DCAT estimates the probability of a near-ceiling score (≥53). BCH, Bolck–Croon–Hagenaars; DCAT, distal categorical outcome.

### 3.6 Complementary configurational analysis

No single condition met the predefined necessity criterion for either high or non-high nurse-perceived nurse–patient relationship quality. For high relationship quality, the parsimonious solution combined membership in the high competency–high attitude profile with a favourable perceived hospital ethical climate (consistency = 0.938; coverage = 0.501). In contrast, non-high relationship quality was associated with multiple configurations under the main calibration. Under the alternative P75/P50/P25 calibration, the high-NPR configuration was retained, whereas the non-high NPR solution converged to the simpler ∼HIGHPROFILE*∼HECH configuration, indicating greater calibration sensitivity for the non-high outcome. Detailed solutions and robustness analyses are provided in the Supporting Information.

## 4 Discussion

This study identified distinct competency–attitude profiles, including aligned and discordant patterns, indicating that nurses’ readiness for secure patient-information handling does not follow a simple high-to-low continuum. These findings extend previous person-centred and variable-centred evidence by showing how nursing informatics competency and information-security attitudes coexist within individual nurses.

### 4.1 Organisational context and workplace correlates of profile membership

Perceived hospital ethical climate showed the most consistent association with profile membership. Nurses perceiving a more favourable ethical climate were more likely to belong to the high competency–high attitude profile than to any of the other profiles. Notably, perceived ethical climate was substantially correlated with both nursing informatics competency and information-security attitudes. The concurrent self-report assessment of these constructs may have introduced shared method variance and thereby strengthened the observed correlations. From a Social Cognitive Theory perspective, this pattern is consistent with the view that individual capabilities and cognitive orientations are embedded within environmental influences (12). In digital nursing practice, an ethically supportive environment may reinforce expectations concerning professional responsibility, confidentiality, and appropriate information handling, providing a context in which informatics competency and security-oriented attitudes are more likely to align (13, 14). However, ethical climate was measured at the individual level and therefore reflects nurses’ perceptions of their organisational environment rather than an objective hospital-level characteristic. Accordingly, the observed association should not be interpreted as evidence of an independent or causal effect on profile membership.

Policy familiarity and reporting-procedure knowledge showed more selective associations with particular profiles. These factors represent relatively proximal workplace resources that may help translate broader organisational expectations into concrete information-security practices (7). Their profile-specific associations suggest that perceived ethical climate and more proximal workplace resources may represent distinct contextual layers relevant to competency–attitude patterns. The limited associations observed for most demographic and professional characteristics further indicate that these patterns may not be readily identified from conventional workforce characteristics alone.

### 4.2 Relational relevance of competency–attitude profiles

The high competency–high attitude profile retained the highest adjusted nurse-perceived nurse–patient relationship quality, although between-profile differences were substantially attenuated after accounting for perceived hospital ethical climate and demographic and professional characteristics. The overall profile difference nevertheless remained significant, suggesting that competency–attitude configurations may retain relational relevance beyond these measured covariates, although the unadjusted differences should not be interpreted as independent effects of profile membership. Digital technologies can support information access and care coordination while also shaping nurses’ attention, communication and responsibility for confidential information (16). Informatics competency without a corresponding security orientation may therefore provide an incomplete basis for secure digital practice, whereas favourable security attitudes may be more difficult to translate into practice when informatics competency is limited.

This interpretation is consistent with the importance of confidentiality, accountability and trust within the nurse–patient relationship (15). Competent and secure information handling may be associated with conditions that support professional nurse–patient interactions in increasingly digital care environments. However, nurse–patient relationship quality was examined as a distal relational outcome rather than a direct consequence of profile membership. The cross-sectional design precludes causal inference, and the measure reflects nurses’ rather than patients’ perceptions. These findings should also be interpreted in light of the fact that all major variables were self-reported by nurses at the same time point, which may have introduced common-method bias and socially desirable responding and thereby strengthened some of the observed associations.

### 4.3 Profile–context configurations and relational quality

The fsQCA findings provide a complementary set-theoretic perspective on profile–context combinations, although its incremental contribution should be interpreted cautiously. The high-NPR configuration combined membership in the high competency–high attitude profile with favourable perceived HEC. Given the strong associations of perceived HEC with both profile membership and NPR, this conjunction is better interpreted as the co-occurrence of closely related conditions than as evidence of independent explanatory domains. The non-high NPR findings provided some indication of configurational asymmetry under the main calibration, but this pattern was less stable under alternative calibration, where the solution simplified substantially. Accordingly, the fsQCA findings should be regarded as exploratory configurational evidence rather than as evidence of causal mechanisms or as validation of the latent profiles (33–36).

### 4.4 Profile-informed strategies for workforce development

The findings suggest the potential value of a profile-informed, differentiated approach to digital workforce development. The observed discordant and broadly lower profiles may inform hypotheses regarding different areas of future workforce support. Specifically, discordance between informatics competency and information-security attitudes may warrant evaluation of support directed toward the comparatively less favourable domain, whereas broadly lower patterns may warrant consideration of support across both domains. These possibilities were not evaluated as interventions in the present study.

At the organisational level, such differentiated support may be reinforced by clear patient-information protection policies, accessible reporting procedures and an ethical climate that positions secure information handling as part of professional nursing practice (3, 7). Profile-informed assessment should not be used to assign fixed labels to nurses, but rather to identify combinations of strengths and support needs that may be obscured by overall scores. Accordingly, these profile-informed strategies should be regarded as potential approaches for future development and evaluation rather than established recommendations for practice.

### 4.5 Strengths and limitations

A key strength of this study was the joint person-centred examination of nursing informatics competency and information-security attitudes, supported by analyses that accounted for classification uncertainty and the ceiling effect in nurse–patient relationship scores.

Several limitations should be considered. The cross-sectional design precludes temporal or causal inference. The single-centre convenience sample from a Grade A tertiary hospital, in which 86.0% of participants had received previous information-security/cybersecurity training, may limit generalisability to nurses in smaller or less digitally mature healthcare settings. All major variables were assessed concurrently using self-report measures, so common-method and social-desirability biases, including shared response tendencies, cannot be excluded and may have strengthened some of the observed associations. Nurse–patient relationship quality reflected nurses’ rather than patients’ perceptions. Perceived hospital ethical climate was assessed at the individual level and should not be interpreted as an objective organisational characteristic. Although the adjusted BCH analysis accounted for perceived hospital ethical climate and selected demographic and professional characteristics, residual confounding cannot be excluded. Latent profiles are sample-dependent, and both the covariance-specification and completion-time sensitivity analyses indicated that finer-grained distinctions among the lower profiles, particularly the C1/C3 distinction, were less stable than the broader aligned and discordant patterns. The fsQCA findings should be regarded as exploratory. Future multicentre and longitudinal studies should examine the reproducibility and stability of the identified competency–attitude patterns and incorporate patient-reported or behavioural outcomes. Intervention studies could further examine whether targeted organisational or educational strategies are associated with changes in these patterns and related outcomes.

## 5 Conclusion

Clinical nurses exhibited distinct aligned and discordant configurations of nursing informatics competency and information-security attitudes, indicating that readiness for secure patient-information handling does not follow a simple high-to-low continuum. A more favourable perceived hospital ethical climate was associated with the high competency–high attitude profile. This profile retained the highest adjusted nurse-perceived nurse–patient relationship quality, although between-profile differences were attenuated after accounting for perceived hospital ethical climate and demographic and professional characteristics. These findings suggest that joint competency–attitude profiles may help inform future workforce development and workplace support for secure digital practice. Replication in multicentre and longitudinal studies is needed before profile-informed approaches can be translated into established practice recommendations.

## Data Availability

The datasets presented in this article are not readily available because of privacy and confidentiality considerations. Access may be considered on reasonable request to the corresponding author, subject to institutional approval and applicable data-protection requirements. Requests to access the datasets should be directed to Dongxia Cao, 285401065@qq.com.

## References

[1] U.S. Department of Health and Human Services, Office for Civil Rights. Annual report to Congress on breaches of unsecured protected health information for calendar year 2024 (2026). Available online at: https://www.hhs.gov/sites/default/files/breach-report-to-congress-2024.pdf (Accessed September 4, 2026). 10.3109/15360288.2015.1037530 26095483

[2] European Parliament and Council of the European Union. Regulation (EU) 2025/327 of 11 February 2025 on the European health data space and amending directive 2011/24/EU and regulation (EU) 2024/2847. Off J Eur Union. (2025). Available online at: http://data.europa.eu/eli/reg/2025/327/oj

[3] National Health Commission of the People’s Republic of China, National Administration of Traditional Chinese Medicine, National Disease Control and Prevention Administration. Measures for cybersecurity management of medical and health institutions (2022).

[4] U.S. Department of Health and Human Services, Office for Civil Rights. HIPAA Security Rule notice of proposed rulemaking to strengthen cybersecurity for electronic protected health information (2024). Available online at: https://www.hhs.gov/hipaa/for-professionals/security/hipaa-security-rule-nprm/index.html (Accessed September 4, 2026)). 10.3109/15360288.2015.1037530 26095483

[5] Kang J Seomun GA. Development and validation of the information security attitude questionnaire (ISA-Q) for nurses. Nurs Open. (2023) 10:850–60. 10.1002/nop2.1353 36058000 PMC9834181

[6] Kinnunen U-M Kuusisto A Koponen S Ahonen O Kaihlanen A-M Hassinen T. Nurses’ informatics competency assessment of health information system usage: a cross-sectional survey. Comput Inform Nurs. (2023) 41:869–76. 10.1097/CIN.0000000000001026 37931302 PMC10662616

[7] Kang P Kang J Monsen KA. Nurse information security policy compliance, information competence, and information security attitudes predict information security behavior. Comput Inform Nurs. (2023) 41:595–602. 10.1097/CIN.0000000000000981 36730714

[8] Chen X Zhu L Gong S He Y Chen H Zhao X. Population characteristics and influencing factors of clinical nurses’ informatics competency: a latent profile analysis. West J Nurs Res. (2025) 47:641–50. 10.1177/01939459251333686 40271955

[9] Kaihlanen A-M Elovainio M Virtanen L Kinnunen U-M Vehko T Saranto K. Nursing informatics competence profiles and perceptions of health information system usefulness among registered nurses: a latent profile analysis. J Adv Nurs. (2023) 79:4022–33. 10.1111/jan.15718 37243421

[10] Wang S Xu J Zhu J Chen J. Clinical nurses’ information security attitude profiles and associated factors [article in Chinese]. Journal of Nursing Administration. (2026) 26(1):1–6. 10.3969/j.issn.1671-315x.2026.01.001

[11] Xue Y Yin C Bai X Ji S Ma F Zheng D. The mediating effect of error management climate on the relationship between nursing informatics competency and information security attitude among clinical nurses: a cross-sectional study. Front Public Health. (2026) 14:1887079. 10.3389/fpubh.2026.1887079 42500015 PMC13395990

[12] Bandura A. Social cognitive theory: an agentic perspective. Annu Rev Psychol. (2001) 52:1–26. 10.1146/annurev.psych.52.1.1 11148297

[13] Essex R Thompson T Evans TR Fortune V Kalocsányiová E Miller D. Ethical climate in healthcare: a systematic review and meta-analysis. Nurs Ethics. (2023) 30:910–21. 10.1177/09697330231177419 PMC10710009 37459590

[14] Park SK Jeong YW. Relationship between hospital ethical climate, critical thinking disposition, and nursing task performance. BMC Nurs. (2024) 23:696. 10.1186/s12912-024-02366-1 39334039 PMC11438314

[15] Allande-Cussó R Fernández-García E Porcel-Gálvez AM. Defining and characterising the nurse–patient relationship: a concept analysis. Nurs Ethics. (2022) 29:462–84. 10.1177/09697330211046651 34879785

[16] Forde-Johnston C Butcher D Aveyard H. An integrative review exploring the impact of electronic health records on the quality of nurse–patient interactions and communication. J Adv Nurs. (2023) 79:48–67. 10.1111/jan.15484 36345050 PMC10100205

[17] Tein JY Coxe S Cham H. Statistical power to detect the correct number of classes in latent profile analysis. Struct Equ Modeling. (2013) 20(4):640–57. 10.1080/10705511.2013.824781 24489457 PMC3904803

[18] Von Elm E Altman DG Egger M Pocock SJ Gøtzsche PC Vandenbroucke JP. The strengthening the reporting of observational studies in epidemiology (STROBE) statement: guidelines for reporting observational studies. Lancet. (2007) 370:1453–57. 10.1016/S0140-6736(07)61602-X 18064739

[19] Leiner DJ. Too fast, too straight, too weird: non-reactive indicators for meaningless data in internet surveys. Surv Res Methods. (2019) 13(3):229–48. 10.18148/srm/2019.v13i3.7403

[20] Greszki R Meyer M Schoen H. Exploring the effects of removing “too fast” responses and respondents from web surveys. Public Opin Q. (2015) 79(2):471–503. 10.1093/poq/nfu058

[21] Cai ZZ Chen Y Yan LJ Zheng W Lin B Wu L. Development of nursing informatics competency assessment scale for clinical nurses: testing of reliability and validity. J Nurs Sci. (2022) 37(24):43–6. 10.3870/j.issn.1001-4152.2022.24.043

[22] Liu MD Ding SN Yang ZC Wang JN Zhou JY Wang JR. Reliability and validity of the Chinese version of the information security attitude questionnaire. J Nurs Sci. (2024) 39(17):15–9. 10.3870/j.issn.1001-4152.2024.17.015 PMC11157161 38845463

[23] Olson LL. Hospital nurses’ perceptions of the ethical climate of their work setting. Image J Nurs Sch. (1998) 30:345–49. 10.1111/j.1547-5069.1998.tb01331.x 9866295

[24] Wang L. Translation and application of the hospital ethical climate survey (Master’s thesis). Zhengzhou University, Zhengzhou, China (2018).

[25] Ma SZ Gou L Zeng WL. Development and validation of the nurse–patient relationship scale from the nurse perspective. J Nurs Sci. (2020) 35(10):55–8. 10.3870/j.issn.1001-4152.2020.10.055

[26] Nylund KL Asparouhov T Muthén BO. Deciding on the number of classes in latent class analysis and growth mixture modeling: a monte carlo simulation study. Struct Equ Modeling. (2007) 14:535–69. 10.1080/10705510701575396

[27] Van Lissa CJ Garnier-Villarreal M Anadria D. Recommended practices in latent class analysis using the open-source R-package tidySEM. Struct Equ Modeling. (2024) 31:526–34. 10.1080/10705511.2023.2250920

[28] Asparouhov T Muthén B. Auxiliary variables in mixture modeling: three-step approaches using mplus. Struct Equ Modeling. (2014) 21:329–41. 10.1080/10705511.2014.915181

[29] Vermunt JK. Latent class modeling with covariates: two improved three-step approaches. Polit Anal. (2010) 18:450–69. 10.1093/pan/mpq025

[30] Bakk Z Vermunt JK. Robustness of stepwise latent class modeling with continuous distal outcomes. Struct Equ Modeling. (2016) 23:20–31. 10.1080/10705511.2014.955104

[31] Bolck A Croon M Hagenaars J. Estimating latent structure models with categorical variables: one-step versus three-step estimators. Polit Anal. (2004) 12:3–27. 10.1093/pan/mph001

[32] Holm S. A simple sequentially rejective multiple test procedure. Scand J Stat. (1979) 6:65–70.

[33] Gabriel AS Campbell JT Djurdjevic E Johnson RE Rosen CC. Fuzzy profiles: comparing and contrasting latent profile analysis and fuzzy set qualitative comparative analysis for person-centered research. Organ Res Methods. (2018) 21:877–904. 10.1177/1094428117752466

[34] Pappas IO Woodside AG. Fuzzy-set qualitative comparative analysis (fsQCA): guidelines for research practice in information systems and marketing. Int J Inf Manag. (2021) 58:102310. 10.1016/j.ijinfomgt.2021.102310

[35] Ragin CC. Redesigning Social Inquiry: Fuzzy Sets and Beyond. Chicago, IL: University of Chicago Press (2008). 10.7208/9780226702797

[36] Thomann E Ege J Paustyan E. Approaches to qualitative comparative analysis and good practices: a systematic review. Swiss Polit Sci Rev. (2022) 28:557–80. 10.1111/spsr.12503

