# Supplementary material for: Aligned and discordant profiles of nursing informatics competency and information-security attitudes among clinical nurses: a single-centre study in China
Source: Front Med (Lausanne). 2026 Oct 14;13:1987978. doi: 10.3389/fmed.2026.1987978 (PMC13713499; doi:10.3389/fmed.2026.1987978)
Supplement: Supplementary file 1 [file Table1.docx]

**Supporting Information**

**Aligned and Discordant Profiles of Nursing Informatics Competency and Information-Security Attitudes Among Clinical Nurses: A Single-Centre Study in China**

**Supplementary Methods**

For fsQCA, the main truth-table thresholds were frequency = 10, consistency = .80 and PRI = .75. Exact calibration anchors, condition coding, and directional expectations for the intermediate solutions are reported in Table S2. Robustness checks used a consistency threshold of .85, a frequency threshold of 15, exclusion of 44 “unsure/prefer not to answer” risk-event responses, and alternative P75/P50/P25 calibration anchors.

**Completion-time threshold and sensitivity analysis**

Of the 1,340 submitted questionnaires, 22 were excluded because consent was not provided and 52 duplicate submissions were removed, leaving 1,266 consented, deduplicated questionnaires for evaluation of completion time. Completion time was treated as a data-quality indicator based on methodological work showing that unusually rapid responding can identify potentially low-quality responses in web surveys (19, 20); these studies support the use of relative completion speed rather than a universal time cutoff. Among the 1,266 questionnaires, the median completion time was 475.5 s (IQR, 331.0–676.3 s), and the 10th percentile was 245 s. The 240-s cutoff therefore lay near the lower 10th percentile, was approximately one-half of the median completion time, and corresponded to an average of less than 2.7 s per substantive scale item across the 90 scale items, even before accounting for demographic and professional questions. The cutoff was therefore treated as a study-specific, empirically anchored data-quality threshold based on questionnaire length and the lower tail of the observed completion-time distribution. To assess sensitivity to this criterion, the 117 submissions completed in <240 s were retained and the fixed six-profile LPA was re-estimated using the same nine indicators and model specification as in the primary analysis. With 10,000 initial-stage and 2,000 final-stage random starts, the sensitivity model yielded an entropy of .952. Four profile patterns corresponding to C2 and C4–C6 were closely reproduced, whereas the primary C1/C3 distinction was not retained. An extreme three-participant class (0.24%) emerged, accompanied by a non-positive definite first-order derivative product matrix warning. Detailed results are summarised in Table S9.

**Table S1. Full R3STEP estimates for correlates of profile membership**

| **Variable** | **C1 vs C4** | **P** | **C2 vs C4** | **P** | **C3 vs C4** | **P** | **C5 vs C4** | **P** | **C6 vs C4** | **P** |
| --- | --- | --- | --- | --- | --- | --- | --- | --- | --- | --- |
| Information-security training | 0.731 (0.311–1.719) | .473 | 1.285 (0.436–3.792) | .649 | 1.659 (0.697–3.944) | .252 | 1.107 (0.470–2.608) | .815 | 0.824 (0.401–1.696) | .600 |
| Policy familiarity | 0.447 (0.289–0.690) | <.001 | 0.562 (0.326–0.969) | .038 | 0.664 (0.446–0.988) | .043 | 0.586 (0.369–0.931) | .024 | 0.805 (0.555–1.168) | .253 |
| Reporting-procedure knowledge | 0.583 (0.404–0.841) | .004 | 0.641 (0.396–1.039) | .071 | 0.742 (0.537–1.025) | .070 | 0.509 (0.348–0.744) | <.001 | 0.870 (0.626–1.210) | .408 |
| Risk-event frequency: occasionally vs never | 0.567 (0.300–1.073) | .081 | 0.858 (0.391–1.883) | .703 | 1.014 (0.562–1.829) | .963 | 1.116 (0.592–2.107) | .734 | 0.735 (0.424–1.275) | .273 |
| Risk-event frequency: sometimes vs never | 0.496 (0.163–1.506) | .216 | 1.242 (0.353–4.372) | .735 | 2.875 (1.102–7.497) | .031 | 1.853 (0.636–5.404) | .258 | 1.079 (0.441–2.637) | .868 |
| Risk-event frequency: frequently vs never | 0.253 (0.009–7.426) | .425 | 0.566 (0.044–7.292) | .663 | 0.799 (0.113–5.630) | .822 | 0.983 (0.112–8.642) | .988 | 0.570 (0.147–2.204) | .415 |
| Risk-event frequency: unsure/prefer not to answer vs never | 0.397 (0.076–2.078) | .274 | 0.778 (0.175–3.458) | .742 | 0.656 (0.170–2.533) | .541 | 0.748 (0.171–3.267) | .700 | 0.342 (0.089–1.311) | .118 |
| Perceived hospital ethical climate | 0.035 (0.022–0.055) | <.001 | 0.081 (0.051–0.131) | <.001 | 0.091 (0.060–0.137) | <.001 | 0.116 (0.077–0.175) | <.001 | 0.153 (0.104–0.225) | <.001 |
| Professional title | 0.829 (0.498–1.381) | .472 | 1.067 (0.541–2.107) | .851 | 1.009 (0.606–1.680) | .972 | 1.511 (0.863–2.647) | .149 | 1.347 (0.898–2.021) | .150 |
| Clinical experience | 1.189 (0.762–1.855) | .446 | 1.142 (0.643–2.028) | .650 | 0.923 (0.602–1.415) | .714 | 0.955 (0.579–1.574) | .857 | 1.175 (0.840–1.642) | .346 |
| Age | 0.972 (0.576–1.641) | .916 | 0.896 (0.485–1.654) | .725 | 0.822 (0.498–1.358) | .444 | 0.795 (0.449–1.410) | .433 | 0.794 (0.530–1.189) | .262 |
| Education | 0.424 (0.227–0.793) | .007 | 1.021 (0.468–2.228) | .958 | 0.684 (0.372–1.258) | .222 | 0.567 (0.287–1.120) | .102 | 0.611 (0.367–1.018) | .058 |

*Note.* C4 (high competency–high attitude) was the reference profile. Values are ORs (95% CIs) from R3STEP multinomial logistic regression; bold indicates P < .05. Training compares yes with no. Risk-event frequency uses never as the reference and retains “unsure/prefer not to answer” as a separate category. Ordinal variables are expressed per category increase, and perceived hospital ethical climate is expressed per 1-SD increase in the final 25-item HECS mean score. OR = odds ratio; CI = confidence interval.

**Table S2. fsQCA calibration and condition coding**

| **Set/condition** | **Type** | **Full membership** | **Crossover** | **Full non-membership / coding** |
| --- | --- | --- | --- | --- |
| HIGHPROFILE | Crisp | 1 = C4 | — | 0 = all other profiles |
| TRAIN | Crisp | 1 = yes | — | 0 = no |
| HECH | Direct fuzzy | 125 | 111 | 96 |
| HNPR | Direct fuzzy | 54 | 50 | 42 |
| POLICYH | Ordered fuzzy | Very familiar = 1.00 | Fairly familiar = 0.67 | Slightly familiar = 0.33; not familiar = 0 |
| REPORTH | Ordered fuzzy | Very clear = 1.00 | Clear = 0.67 | Somewhat clear = 0.33; unclear = 0 |
| LOWRISK | Ordered fuzzy | Never = 1.00 | Unsure/prefer not to answer = 0.50 | Occasionally = 0.33; sometimes = 0.17; frequently = 0 |

*Note.* HECH and HNPR used direct calibration. Primary anchors were empirically selected from the observed distributions using the 90th percentile, median, and 10th percentile for full membership, crossover, and full non-membership, respectively. For HNPR, the 90th percentile corresponded to the scale maximum of 54. Directional expectations (HIGHPROFILE, HECH, TRAIN, POLICYH, REPORTH, LOWRISK) were (1, 1, 1, 1, 1, 1) for HNPR and (0, 0, 0, 0, 0, 0) for ~HNPR. Alternative P75/P50/P25 anchors were 123/111/100 for HECH and 54/50/45 for HNPR.

**Table S3. fsQCA configurations associated with high and non-high nurse–patient relationship quality**

| **Outcome** | **Solution** | **Configuration** | **Consistency** | **PRI** | **Raw coverage** | **Unique coverage** |
| --- | --- | --- | --- | --- | --- | --- |
| High NPR | Parsimonious P1 | HIGHPROFILE*HECH | .938 | .930 | .501 | — |
| High NPR | Intermediate H1 | HIGHPROFILE*HECH*TRAIN*POLICYH*REPORTH | .943 | .934 | .396 | .059 |
| High NPR | Intermediate H2 | HIGHPROFILE*HECH*TRAIN*POLICYH*LOWRISK | .950 | .942 | .355 | .018 |
| High NPR | Intermediate H3 | HIGHPROFILE*HECH*POLICYH*REPORTH*LOWRISK | .954 | .947 | .355 | .018 |
| High NPR | Overall intermediate | H1 + H2 + H3 | .938 | .930 | .432 | — |
| Non-high NPR | Intermediate I1-1 | ~HECH*~REPORTH | .852 | .775 | .469 | .109 |
| Non-high NPR | Intermediate I1-2 | ~HIGHPROFILE*~HECH*TRAIN | .812 | .760 | .630 | .208 |
| Non-high NPR | Intermediate I1-3 | ~HIGHPROFILE*~HECH*POLICYH*~LOWRISK | .886 | .840 | .259 | .007 |
| Non-high NPR | Overall intermediate I1 | I1-1 + I1-2 + I1-3 | .810 | .750 | .775 | — |
| Non-high NPR | Intermediate I2-1 | ~HIGHPROFILE*~HECH*TRAIN | .812 | .760 | .630 | .236 |
| Non-high NPR | Intermediate I2-2 | ~HIGHPROFILE*~HECH*~POLICYH*~REPORTH | .892 | .829 | .327 | .012 |
| Non-high NPR | Intermediate I2-3 | ~HIGHPROFILE*~HECH*~REPORTH*LOWRISK | .866 | .785 | .351 | .014 |
| Non-high NPR | Intermediate I2-4 | ~HIGHPROFILE*~HECH*POLICYH*REPORTH*~LOWRISK | .893 | .842 | .221 | .008 |
| Non-high NPR | Overall intermediate I2 | I2-1 + I2-2 + I2-3 + I2-4 | .816 | .763 | .735 | — |

*Note.* The main truth-table thresholds were frequency = 10, consistency = .80 and PRI = .75. No single condition reached the predefined necessity threshold of .90. HIGHPROFILE = membership in C4 (high competency–high attitude); HECH = high perceived hospital ethical climate; TRAIN = information-security/cybersecurity training; POLICYH = high policy familiarity; REPORTH = high reporting-procedure knowledge; LOWRISK = low information-security risk-event frequency; ~ indicates condition absence; PRI = proportional reduction in inconsistency. Non-high NPR yielded multiple equivalent parsimonious solutions; the two intermediate solution forms are presented here.

**Table S4. Robustness analyses of fsQCA results**

| **Outcome** | **Robustness check** | **Configuration/solution** | **Consistency** | **PRI** | **Coverage** | **Interpretation** |
| --- | --- | --- | --- | --- | --- | --- |
| High NPR | Main analysis | HIGHPROFILE*HECH | .938 | .930 | .501 | Baseline parsimonious solution |
| High NPR | Consistency threshold = .85 | HIGHPROFILE*HECH | .938 | .930 | .501 | Core configuration retained |
| High NPR | Frequency threshold = 15 | HIGHPROFILE*HECH | .938 | .930 | .501 | Core configuration retained |
| High NPR | Excluded 44 unsure/prefer-not-to-answer responses | HIGHPROFILE*HECH | .940 | .932 | .498 | Core configuration retained |
| High NPR | Alternative P75/P50/P25 calibration | HIGHPROFILE*HECH | .918 | .909 | .528 | Core configuration retained |
| Non-high NPR | Main calibration | Intermediate I1 / I2 | .810 / .816 | .750 / .763 | .775 / .735 | Multiple configurations |
| Non-high NPR | Excluded 44 unsure/prefer-not-to-answer responses | Intermediate I1 / I2 | .810 / .815 | .751 / .762 | .776 / .738 | Pattern retained |
| Non-high NPR | Alternative P75/P50/P25 calibration | ~HIGHPROFILE*~HECH | .825 | .796 | .764 | Converged to a simpler solution |

*Note.* For high NPR, consistency, PRI and coverage refer to the parsimonious solution. For non-high NPR under the main and unsure/prefer-not-to-answer response-exclusion analyses, values correspond to the two alternative intermediate solution forms (I1 / I2). Alternative calibration used P75/P50/P25 anchors. The high-NPR parsimonious configuration HIGHPROFILE*HECH remained present across all reported robustness checks, whereas non-high NPR was more calibration-sensitive.

**Table S5. Standardised means of the nine LPA indicators across the six profiles**

| **Indicator** | **C1** | **C2** | **C3** | **C4** | **C5** | **C6** |
| --- | --- | --- | --- | --- | --- | --- |
| NIC-C | -1.202 | -0.501 | -0.023 | 0.948 | -0.434 | -0.349 |
| NIC-I | -1.076 | -0.665 | 0.174 | 1.004 | -0.492 | -0.570 |
| NIC-A | -1.272 | -0.570 | -0.076 | 1.001 | -0.421 | -0.344 |
| ISA-EC | -0.880 | 0.107 | -0.999 | 0.559 | -0.589 | 0.466 |
| ISA-FS | -1.019 | -0.066 | -0.950 | 0.671 | -0.798 | 0.480 |
| ISA-AR | -0.961 | -0.230 | -0.897 | 0.673 | -0.734 | 0.421 |
| ISA-WS | -1.380 | 0.057 | -1.283 | 0.811 | -0.842 | 0.676 |
| ISA-PR | -1.339 | -0.329 | -1.214 | 0.772 | -0.393 | 0.598 |
| ISA-CE | -1.480 | -0.831 | -1.406 | 0.732 | 0.401 | 0.685 |

*Note.* Values are profile-specific means of the z-standardised indicators (0 = overall sample mean). C1 = low competency–low attitude; C2 = competency-lagged, attitude-mixed; C3 = attitude-lagged, competency-preserved; C4 = high competency–high attitude; C5 = continuing-education-oriented lower profile; C6 = attitude-advantaged, competency-lagged. NIC-C = computer competence; NIC-I = information competence; NIC-A = comprehensive application competence; ISA-EC = environmental control; ISA-FS = facility stability; ISA-AR = access restriction; ISA-WS = work systematization; ISA-PR = professional responsibility; ISA-CE = continuing education.

**Table S6. Descriptive statistics and Spearman rank correlations among study variables (n = 1,149)**

| **Variable** | **Median (P25, P75)** | **1** | **2** | **3** | **4** |
| --- | --- | --- | --- | --- | --- |
| 1. NIC | 101.00 (82.00, 120.00) | 1 | — | — | — |
| 2. ISA | 112.00 (94.00, 119.00) | 0.555 | 1 | — | — |
| 3. HEC | 111.00 (100.00, 123.00) | 0.672 | 0.620 | 1 | — |
| 4. NPR | 50.00 (45.00, 54.00) | 0.557 | 0.549 | 0.746 | 1 |

*Note.* P25 and P75 denote the 25th and 75th percentiles. Values below the diagonal are Spearman rank correlation coefficients; all correlations were significant at P < .001. NIC = nursing informatics competency; ISA = information-security attitudes; HEC = perceived hospital ethical climate; NPR = nurse-perceived nurse–patient relationship quality.

**Table S7. Internal consistency and intercorrelations of the nine LPA indicators (n = 1,149)**

| **Indicator** | **Cronbach’s α** | **1** | **2** | **3** | **4** | **5** | **6** | **7** | **8** | **9** |
| --- | --- | --- | --- | --- | --- | --- | --- | --- | --- | --- |
| 1. NIC-C | 0.912 | 1 |  |  |  |  |  |  |  |  |
| 2. NIC-I | 0.965 | .776 | 1 |  |  |  |  |  |  |  |
| 3. NIC-A | 0.958 | .745 | .820 | 1 |  |  |  |  |  |  |
| 4. ISA-EC | 0.754 | .364 | .267 | .368 | 1 |  |  |  |  |  |
| 5. ISA-FS | 0.821 | .424 | .362 | .452 | .689 | 1 |  |  |  |  |
| 6. ISA-AR | 0.779 | .455 | .393 | .434 | .558 | .662 | 1 |  |  |  |
| 7. ISA-WS | 0.953 | .528 | .447 | .562 | .659 | .738 | .719 | 1 |  |  |
| 8. ISA-PR | 0.887 | .506 | .432 | .528 | .605 | .678 | .657 | .842 | 1 |  |
| 9. ISA-CE | 0.947 | .494 | .403 | .528 | .581 | .639 | .629 | .841 | .836 | 1 |

***Note.*** Cronbach’s α values are dimension-specific estimates for the present sample. Values below the diagonal are Pearson correlations; all P < .001. NIC-C = computer competence; NIC-I = information competence; NIC-A = comprehensive application competence; ISA-EC = environmental control; ISA-FS = facility stability; ISA-AR = access restriction; ISA-WS = work systematization; ISA-PR = professional responsibility; ISA-CE = continuing education.

**Table S8. Sensitivity analysis of the retained six-profile solution under a less restrictive within-profile covariance specification**

| **Characteristic** | **Primary model** | **Sensitivity model** |
| --- | --- | --- |
| Number of profiles | 6 | 6 |
| Within-profile residual covariances | Fixed at zero | Four class-invariant residual covariances freely estimated |
| Initial/final random starts | 5,000 / 1,000 | 5,000 / 1,000 |
| Best log-likelihood | −9744.622 | −9461.700 |
| Best log-likelihood replications | 215 | 361 |
| AIC | 19625.244 | 19067.400 |
| BIC | 19968.416 | 19430.759 |
| aBIC | 19752.427 | 19202.064 |
| Entropy | .939 | .916 |
| Smallest profile, n (%) | 61 (5.31%) | 57 (4.96%) |
| Modal-classification agreement | — | 74.2% |
| Adjusted Rand index | — | .673 |
| Interpretation | Retained six-profile solution | Partial robustness; C1 and C3 largely merged, and C6 showed some redistribution |

*Note.* The sensitivity model freed the following class-invariant within-profile residual covariances: NIC-I WITH NIC-A, ISA-WS WITH ISA-PR, ISA-WS WITH ISA-CE, and ISA-PR WITH ISA-CE. Modal-classification agreement and the adjusted Rand index compare the sensitivity model with the primary six-profile solution after class alignment. AIC = Akaike information criterion; BIC = Bayesian information criterion; aBIC = sample-size-adjusted Bayesian information criterion.

**Table S9. Sensitivity analysis of the 240-s completion-time exclusion criterion**

| **Characteristic** | **Primary analysis** | **Sensitivity analysis retaining <240-s submissions** |
| --- | --- | --- |
| Sample size, n | 1,149 | 1,266 |
| Completion-time criterion | <240 s excluded | <240 s retained |
| Number of profiles | 6 | 6 (fixed) |
| LPA indicators | Nine | Same nine indicators |
| Model specification | Primary six-profile specification | Same as primary analysis |
| Initial/final random starts | 5,000 / 1,000 | 10,000 / 2,000 |
| Best log-likelihood | −9744.622 | −10695.550 |
| Best log-likelihood replications | 215 | 6 |
| Entropy | 0.939 | 0.952 |
| C1, n (%) | 183 (15.93) | 3 (0.24) |
| C2, n (%) | 61 (5.31) | 64 (5.06) |
| C3, n (%) | 139 (12.10) | 353 (27.88) |
| C4, n (%) | 408 (35.51) | 480 (37.91) |
| C5, n (%) | 96 (8.36) | 102 (8.06) |
| C6, n (%) | 262 (22.80) | 264 (20.85) |
| Modal-classification agreement among common respondents | — | 82.3% |
| Adjusted Rand index among common respondents | — | .848 |
| Estimation warning | None | Non-positive definite first-order derivative product matrix involving the three-participant class |
| Interpretation | Retained six-profile solution | Four primary profile patterns closely reproduced; primary C1/C3 distinction not retained |

Note. The sensitivity analysis retained the 117 submissions excluded from the primary analysis solely because completion time was <240 s, while preserving the other exclusion criteria. The six-profile model was re-estimated as a fixed sensitivity model rather than repeating profile enumeration. Sensitivity classes were aligned to the closest primary-profile patterns for descriptive comparison. Four profile patterns corresponding to C2 and C4–C6 were closely reproduced, whereas the primary C1/C3 distinction was not retained. The three-participant C1-aligned sensitivity class should not be interpreted as a substantive profile. Mplus reported a non-positive definite first-order derivative product matrix involving this class. Modal-classification agreement and the adjusted Rand index were calculated among the 1,149 respondents common to both analyses after class alignment. Increasing random starts from 5,000/1,000 to 10,000/2,000 reproduced the same sensitivity solution.

**Supplementary Mplus Syntax**

The following syntax reproduces the retained six-profile high-start stability check and the less restrictive six-profile sensitivity model. The indicator order was v1 = NIC-C, v2 = NIC-I, v3 = NIC-A, v4 = ISA-EC, v5 = ISA-FS, v6 = ISA-AR, v7 = ISA-WS, v8 = ISA-PR, and v9 = ISA-CE.

**Primary retained six-profile model (high-start stability check)**

```
TITLE: Population-SD standardized 9-indicator LPA 6-class high-start stability check;
DATA: FILE IS LPA_9dims_population_z.dat;
VARIABLE:
  NAMES ARE v1 v2 v3 v4 v5 v6 v7 v8 v9;
  USEVARIABLES ARE v1 v2 v3 v4 v5 v6 v7 v8 v9;
  CLASSES = c(6);
  MISSING ARE ALL (-999);
ANALYSIS:
  TYPE = MIXTURE;
  ESTIMATOR = MLR;
  STARTS = 5000 1000;
  STITERATIONS = 20;
  LRTSTARTS = 0 0 500 100;
MODEL:
OUTPUT: TECH11 TECH14;
```

**Less restrictive six-profile sensitivity model**

```
TITLE: Population-SD LPA 6-class sensitivity with four class-invariant residual covariances;
DATA: FILE IS LPA_9dims_population_z.dat;
VARIABLE:
  NAMES ARE v1 v2 v3 v4 v5 v6 v7 v8 v9;
  USEVARIABLES ARE v1 v2 v3 v4 v5 v6 v7 v8 v9;
  CLASSES = c(6);
  MISSING ARE ALL (-999);
ANALYSIS:
  TYPE = MIXTURE;
  ESTIMATOR = MLR;
  STARTS = 5000 1000;
  STITERATIONS = 20;
  LRTSTARTS = 0 0 500 100;
MODEL:
  %OVERALL%
  v2 WITH v3;
  v7 WITH v8 v9;
  v8 WITH v9;
OUTPUT: TECH11 TECH14;
```

*Note.* The one- to eight-profile enumeration models used 1,000 initial-stage random starts and 250 final-stage optimisations; the syntax above shows the subsequent high-start re-estimation of the retained six-profile solution. In the primary model, indicator-specific variances were constrained to equality across profiles and within-profile covariances were fixed at zero. The sensitivity model freely estimated the four residual covariances shown above and constrained them to be class-invariant.
